# Various perspectives of “General Medicine” in Japan—Respect for and cooperation with each other as the same “General Medicine Physicians”

**DOI:** 10.1002/jgf2.500

**Published:** 2021-11-01

**Authors:** Yuya Yokota, Takashi Watari

**Affiliations:** ^1^ Family Practice Center of Okayama Okayama Japan; ^2^ Department of General Medicine Okayama University Graduate School of Medicine, Dentistry and Pharmaceutical Sciences Okayama Japan; ^3^ General Medicine center Shimane University Hospital Shimane Japan

## Abstract

In Japan, the general medicine category includes various specialties: “family physician,” “hospitalist,” and “hospital family physician.” These specialties can be illustrated from two perspectives for an easy understanding of their characteristics.

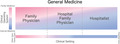

In Japan, the general medicine category includes various specialties, and there is no precise consensus on its classification. Many expressions refer to practicing physicians as “general medicine physicians,” confusing both lay people and medical professionals. As a result, it is a challenge to easily explain the role of general medicine physicians to medical students and young doctors.

In Japan, the specialties of general medicine include “family physician,” “hospitalist,” and “hospital family physician.” These specialties can be illustrated from two perspectives for an easy understanding of their characteristics (Figure 1).

**FIGURE 1:**
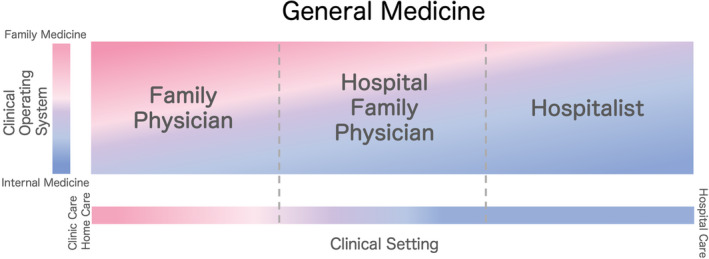
The specialties of general medicine in Japan illustrated from the two perspectives

The role of a general medicine physician changes according to the demands and requirements of each clinical setting; creating clear boundaries is impossible. Therefore, from the first perspective, we devised a scale based on the differences in clinical settings, such as rural areas, clinics, city hospitals, and university hospitals.

From the second but more important perspective, we devised a method to show the ratio of family medicine practices to internal medicine practices. This is very relevant to the thought process and management of the practice, which we have described as the clinical operating system.

Since drawing a clear line between specialties is difficult, it is necessary to express them in gradations from each perspective.

Family physicians provide home care and outpatient care in clinics, not hospitals. They mainly practice family medicine, rather than internal medicine, and play a central role in primary care in the community. Although physicians who provide primary care in the community at their clinics and practice general medicine do not call themselves family physicians, they can be considered so. The role of family physicians in Japan is equivalent to that of general practitioners in the United Kingdom.^1^

Hospitalists mainly practice in medium and large hospitals and do not provide home care. Unlike hospitalists in the United States,^2^ hospitalists in Japan provide inpatient and outpatient care. They mainly practice internal medicine and contribute to medical safety management, postgraduate clinical education, quality of medical care in the hospital, and acute care with a focus on disease management.^3^

Hospital family physicians are family physicians who practice primarily in hospitals. Like hospitalists, they mainly provide inpatient and outpatient care and, occasionally, home care. This style of practice combines those of family physician and hospitalist. Hospital family physicians work in community hospitals that meet a wide range of community health needs and provide inpatient care in all phases ranging from acute to chronic.^4^ Appropriate collaboration with primary care physicians in the community helps to provide comprehensive and continuous outpatient care.

Family physicians and hospital family physicians can be distinguished by their clinical setting. More hospital family physicians are practicing internal medicine than family physicians. Hospitalists and hospital family physicians provide both inpatient and outpatient care, but the latter practice family medicine more consciously. Hospitalists, by contrast, are involved in organizational management to solve hospital problems and hospital improvement, in addition to practicing internal medicine.

Sometimes, the term “general internal medicine” is confused with general medicine in Japan. Many physicians who call themselves “general internists” practice high‐quality general medicine. Similarly, the term “hospitalist” is sometimes used to mean “general internist.” Academically, general internal medicine does not include family medicine.^5^ However, there is little clinical significance in separating general medicine and general internal medicine for patients and medical professionals.

We have looked at how the general medicine specialties in Japan differ from two perspectives. However, although the practice of each specialty varies to some extent, family physicians, hospitalists, and hospital family physicians all share the common practice of responding flexibly to the needs of their respective clinical settings. Therefore, while each specialty has its own identity, it is essential for its members to respect each specialty and cooperate with one another as general medicine physicians. In addition, it is important for these physicians to cooperate with not only specialists but also other generalists, such as home‐visiting physicians, emergency physicians, intensivists, pediatricians, geriatricians, and palliative care physicians, with equal respect for each other.

## CONFLICT OF INTEREST

The authors have stated explicitly that there are no conflicts of interest in connection with this article.
